# Harmonising and linking biomedical and clinical data across disparate data archives to enable integrative cross-biobank research

**DOI:** 10.1038/ejhg.2015.165

**Published:** 2015-08-26

**Authors:** Ola Spjuth, Maria Krestyaninova, Janna Hastings, Huei-Yi Shen, Jani Heikkinen, Melanie Waldenberger, Arnulf Langhammer, Claes Ladenvall, Tõnu Esko, Mats-Åke Persson, Jon Heggland, Joern Dietrich, Sandra Ose, Christian Gieger, Janina S Ried, Annette Peters, Isabel Fortier, Eco JC de Geus, Janis Klovins, Linda Zaharenko, Gonneke Willemsen, Jouke-Jan Hottenga, Jan-Eric Litton, Juha Karvanen, Dorret I Boomsma, Leif Groop, Johan Rung, Juni Palmgren, Nancy L Pedersen, Mark I McCarthy, Cornelia M van Duijn, Kristian Hveem, Andres Metspalu, Samuli Ripatti, Inga Prokopenko, Jennifer R Harris

**Affiliations:** 1 Department of Medical Epidemiology and Biostatistics, Swedish e-Science Research Centre, Karolinska Institutet, Stockholm, Sweden; 2 Department of Pharmaceutical Biosciences and Science for Life Laboratory, Uppsala University, Uppsala, Sweden; 3 European Molecular Biology Laboratory, European Bioinformatics Institute (EMBL-EBI), Wellcome Trust Genome Campus, Hinxton, UK; 4 Uniquer Sarl, rue de la Mercerie, Lausanne, Switzerland; 5 Institute for Molecular Medicine Finland, FIMM, University of Helsinki, Biomedicum Helsinki 2U, Helsinki, Finland; 6 Institute of Epidemiology II, Helmholtz Zentrum München, Deutsches Forschungszentrum für Gesundheit und Umwelt (GmbH), Neuherberg, Germany; 7 Research Unit of Molecular Epidemiology, Helmholtz Zentrum München, Deutsches Forschungszentrum für Gesundheit und Umwelt (GmbH), Neuherberg, Germany; 8 Department of Public Health and General Practice, HUNT Research Centre, Norwegian University of Science and Technology, Levanger, Norway; 9 Department of Clinical Sciences, Diabetes and Endocrinology, Lund University, Lund, Sweden; 10 Lund University Diabetes Centre, CRC at Skåne University Hospital, Malmö, Sweden; 11 Estonian Genome Center, University of Tartu, Tartu, Estonia; 12 Institute of Genetic Epidemiology, Helmholtz Zentrum München, Deutsches Forschungszentrum für Gesundheit und Umwelt (GmbH), Neuherberg; 13 McGill University Health Centre, Montreal, Quebec, Canada; 14 Department of Biological Psychology, FGB, VU University, Amsterdam, The Netherlands; 15 Latvian Genome Data Base (LGDB), Latvian Biomedical Research and Study Centre, Ratsupites 1 k-1, Riga, Latvia; 16 BBMRI-ERIC, Neue Stiftingtalstrasse 2/B/6, Graz, Austria; 17 National Institute for Health and Welfare, Helsinki, Finland; 18 University of Jyvaskyla, Jyväskylä, Finland; 19 Department of Immunology, Genetics and Pathology, Uppsala University, Uppsala, Sweden; 20 Oxford Centre for Diabetes, Endocrinology and Metabolism, University of Oxford, Churchill Hospital, Headington, Oxford, UK; 21 Wellcome Trust Centre for Human Genetics, University of Oxford, Oxford, UK; 22 Oxford NIHR Biomedical Research Centre, Churchill Hospital, Headington, Oxford, UK; 23 Department of Epidemiology, Erasmus Medical Center, Rotterdam, The Netherlands; 24 Department of Public Health, Faculty of Medicine, University of Helsinki, Helsinki, Finland; 25 Wellcome Trust Sanger Institute, Hinxton, Cambridge, UK; 26 Department of Genomics of Common Disease, School of Public Health, Imperial College London, London, UK; 27 Division of Epidemiology, Department of Genes and Environment, The Norwegian Institute of Public Health, Oslo, Norway

## Abstract

A wealth of biospecimen samples are stored in modern globally distributed biobanks. Biomedical researchers worldwide need to be able to combine the available resources to improve the power of large-scale studies. A prerequisite for this effort is to be able to search and access phenotypic, clinical and other information about samples that are currently stored at biobanks in an integrated manner. However, privacy issues together with heterogeneous information systems and the lack of agreed-upon vocabularies have made specimen searching across multiple biobanks extremely challenging. We describe three case studies where we have linked samples and sample descriptions in order to facilitate global searching of available samples for research. The use cases include the ENGAGE (European Network for Genetic and Genomic Epidemiology) consortium comprising at least 39 cohorts, the SUMMIT (surrogate markers for micro- and macro-vascular hard endpoints for innovative diabetes tools) consortium and a pilot for data integration between a Swedish clinical health registry and a biobank. We used the Sample avAILability (SAIL) method for data linking: first, created harmonised variables and then annotated and made searchable information on the number of specimens available in individual biobanks for various phenotypic categories. By operating on this categorised availability data we sidestep many obstacles related to privacy that arise when handling real values and show that harmonised and annotated records about data availability across disparate biomedical archives provide a key methodological advance in pre-analysis exchange of information between biobanks, that is, during the project planning phase.

## Introduction

Biological resources, such as cells, tissues, or biomolecules, are considered to be the essential raw material for the advancement of biotechnology, human health and for research and development in life sciences (see Table 1 for the terminology used in this manuscript).^1^ These biological resources are stored in biobanks and are annotated with digitalised information about the study subjects such as health status, nutrition, lifestyle and environmental exposure. In recent years, biobank-based studies of genetic and molecular factors predisposing to disease, as well as studies of interactions between genetic and environmental or lifestyle factors, have gained momentum.^2^ Meta-analysis techniques have been used to increase sample size and thereby the power to identify genomic regions associated with a variety of clinical outcomes.^3^ However, researchers trying to integrate information across sample collections in the planning phase of a cross-biobank research project face an unprecedented burden of data management tasks. These include the following: (a) determining the types of phenotypic data and biospecimens that are available for research and (b) quantifying the corresponding sample sizes. In practice, retrieving data collected by multiple biobanks over decades is not a trivial task. The white paper ‘Creating a global alliance to enable responsible sharing of genomic and clinical data'^1, 4^ presents many of the challenges for such biomedical data integration such as harmonisation and data security, and pinpoints important issues for the information flow in cross-biobank studies such as data heterogeneity and the lack of harmonised access policies.

Every biobank has internal standards for record keeping, quality assurance and medical procedures. Furthermore, of particular relevance in pan-European and trans-ethnic studies, most of the semantic information is captured in a national language; thus, translation is required for an international research project.^2, 5, 6^ Data heterogeneity is usually addressed on a project-by-project basis: first, the aims of a research project are defined and then biobanks identify and extract relevant data from their internal databases. The design of the project delineates variables of interest (VOIs), which may be different from the variables recorded by the original questionnaires and measurement protocols followed during sample collection. The process of assessing how well a biobank variable is suited for a meta-analysis project and combining data sets wherever variables are considered to be comparable is known as transformation, harmonisation or mapping. In prospective harmonisation, investigators from several research projects will agree on a core set of variables before data collection,^3, 7^ whereas retrospective harmonisation targets synthesis of information already collected by existing legacy studies.^8^ Specific standardised measures have been developed by a number of international organisations, including ILO, UNESCO, OECD and WHO, to facilitate research involving cross-national comparisons with varying scope and success in implementation. Addressing the many challenges requires that researchers have proper knowledge and resources to help them easily, but formally and explicitly, achieve data harmonisation and integration processes that are scientifically valid and replicable,^9^ and that lead to implementation of adequate software solutions.

Access to the actual data about biomaterials in biobank collections, including tests on the samples that result in clinical or epidemiological data, needs to comply with the legal requirements of the country in which the biobank is placed and to the ethical protocols of the organisations involved (http://www.hsern.eu).^2, 10^ This makes it impossible to allow indiscriminate online access to actual data on VOIs. However, these privacy issues can be sidestepped in the initial phase of study design, if biobanks are able to provide information about availability of samples rather than complete sample data online.

Current online information systems for biobanks are typically built as ‘catalogues', where users can query for available sample collections based on a general description of the collection content and obtain summary statistics with the total number of observations and available variables. Such systems lack information on potential availability of variable values sample-by-sample and variable-by-variable. Examples of major biobank catalogues include the EuroBioBank catalogue (http://www.eurobiobank.org/en/services/CatalogueHome.html), the BBMRI (Biobanking and BioMolecular Resource Infrastructure) catalogue of European biobanks (https://www.bbmriportal.eu) and the BBMRI-LPC (The Biobanking and Biomolecular Resources Research Infrastructure–Large Prospective Cohorts) catalogue (http://mineral.iarc.fr). BBMRI-LPC provides a detailed catalogue of the resources that are available within the participating cohorts of the BBMRI-LPC and is an extension of the BBMRI biobank catalogue.

GWAS Central (http://www.gwascentral.org) is a portal for querying a large collection of genetic association studies for summary-level findings. The BioSample database^11^ contains information about biological samples, in particular samples referenced from other databases at the European Bioinformatics Institute. A recent initiative to standardise biobank data sharing is MIABIS 1.0 (Minimum Information About BIobank data Sharing), which describes the data elements that are considered common for all biobanks.^12^ It operates on the level of aggregated biobank information and hence does not offer accurate estimates of sample availability in the context of research questions, but is rather targeted towards higher-level overviews. Queries such as ‘For how many DNA samples in which cohorts are there Type 2 Diabetes status records, as well as fasting glucose concentration and body-mass index?' are not executable in such catalogues.

At the moment, online data resources serving biobank information typically only offer a binary choice between a data access scheme that is ‘open to all' and one that is ‘highly restricted'. There is a lack of reliable systems that provide the essential information across biobanks for planning project design and conducting power calculations that are required for successful grant applications. This creates an obstacle within a research workflow of large meta-studies, that is, 50 000–100 000 samples from multiple collections, in which the processing of data access applications often takes longer than the data analysis itself.

The aim of our study was to demonstrate practical applications of a formalised methodological framework for integration of data across biobanks, which, despite numerous community efforts, developed software and established catalogues, has not been published. To achieve this aim, we present the sample availability (SAIL) method. SAIL operates on availability data (ie, data about data or metadata): information is provided for each sample regarding whether a value for a given phenotypic or genotypic variable exists or not without disclosing the value *per se*, thus allowing researchers to temporarily ignore privacy issues. The method is particularly useful at the onset of large-scale omics studies to investigate specific research questions as well as in raising awareness among researchers in general about the content of biobank data by making the data easier to locate, interpret and incorporate into the design of research studies. Later, in the data analysis part of the project, when real data are to be exchanged, the power calculations conducted in the planning phase with SAIL help generate a detailed study description that may be used in the submission of the application for data access to the relevant ethical committees.

We demonstrate applications of SAIL for data harmonisation and linking using three scenarios of international collaborations: (i) integration of sample information on 39 cohorts in the ENGAGE consortium, (ii) integration of sample information on 15 sample collections in the SUMMIT (surrogate markers for micro- and macro-vascular hard endpoints for innovative diabetes tools) consortium and (iii) a pilot for data integration between a Swedish clinical health registry and a biobank. In this study we start by describing the SAIL method, proceed to describe the three case studies and conclude with discussing the advantages of the method and future directions.

## Methods

We have developed a method to address the issues of retrospective data harmonisation and querying of data about samples across biobanks. The method comprises the following:

- Two data formats for capturing the harmonised data,
- A process for data harmonisation

and it was practically implemented using a software application (SAIL) to make the integrated availability data searchable and accessible online.

### SAIL software application

In an earlier publication, an information system for availability data integration has already been described. The SAIL software package^13^ is a web-based system that provides (1) an interface for harmonisation and submission of sample and phenotype information that is available in various collections, and (2) a search engine for surveying which data from which cohorts could be combined for specific tasks. Rather than presenting the summary content for each collection, it allows resource discovery across biobanks at the level of individual records. Owing to the links between synonymous variables, for example, similar but not equivalent measurements, and to the annotation structure (time point, type of measurements, etc), samples can be searched for by variable, for example, ‘glucose', as well as by a more specific statement, for example, ‘fasting glucose'. For more information about the SAIL data format and software, see Supplementary S4.

### Data formats

Harmonisation in the SAIL method concerns two levels of data:

- Metadata or ‘vocabularies' – collections of terms that are specific to a research project (medical topic) or to a collection of samples. Different types of sample collections, studies and even different users may apply disparate terms when describing the samples.

- Data or ‘samples' – an index of sample IDs by terms stored in vocabularies. Indexing of samples that are available across various resources is essential for effective cross-biobanking research project design, for example, to estimate accurately the number of samples available for a given multi-biobank project. In the SAIL method, indexing can be done using either harmonised or original terms.

Two formats are required for effective communication between IT specialists, data managers and clinicians throughout the process of harmonisation. (1) Vocabularies are taxonomically structured sets of parameters that are used for annotating samples (see example in Supplementary S1). Harmonised and original variables are mapped between vocabularies and either can be used for annotation of samples. The grammar for description of terms is universal and allows for linking terms across vocabularies or studies. In this manner, external shared vocabularies and ontologies can be integrated with internal biobank-specific vocabularies. Examples of relevant external ontologies include the Gene Ontology (GO)^14^, the Phenotype and Trait Ontology and the Human Phenotype Ontology.^15^ (2) The data and availability information format is equally suitable for sample data collection or for sample availability information. In the first case, the matrix of Sample IDs (rows) *vs* Harmonised Terms (columns) is filled with actual parameter values (see Listing F2 in Supplementary S1, MetS:BP and MetS:GLUTM.concentration). In the second case, that is, when only availability data can be collected, the matrix contains ‘0' for ‘value is not recorded' and ‘1' for ‘value is available' for each sample–parameter pair.

### Data harmonisation process

Data harmonisation within the SAIL method consists of the following steps:

Creation of a harmonised vocabulary (HV) for VOIs.
Mapping the HV to the original biobank variables.
Integrating information on the presence of VOIs values for each sample.

**Figure 1:**
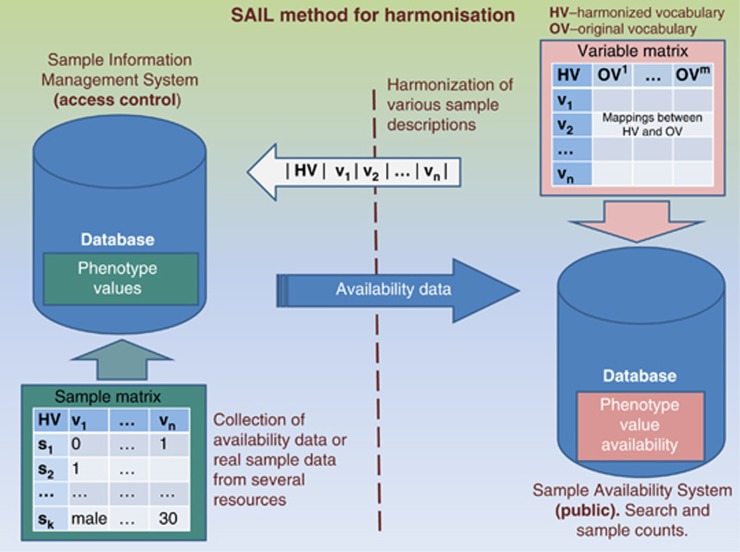
Data harmonisation proceeds on two levels: first, indexing of biospecimens in harmonised terms and, second, harmonisation of variables and descriptors. The left side of the image shows the process of collecting sample information or sample availability information from several resources, that is,. from biobanks, into a database. The right side of the image shows the format for such data submission, defined by harmonising variables. So-called ‘original' vocabularies are descriptors and terms that are used for annotating samples at the biobanks and collections (for the format, see the Methods section). ‘Harmonised' vocabularies are used as common representation of several varieties of original sample descriptors and these are used as submission format and as a configuration of an online resource discovery tool, Sample Availability Information System.

Original variables, that is, those for which values are recorded during collection of biospecimens, as well as harmonised variables, that is, those that are used in a research project later on, are organised as controlled vocabularies or taxonomic structures and stored in an information system. An overview of the phases of data harmonisation in the context of SAIL is illustrated in Figure 1.

**Figure 2:**
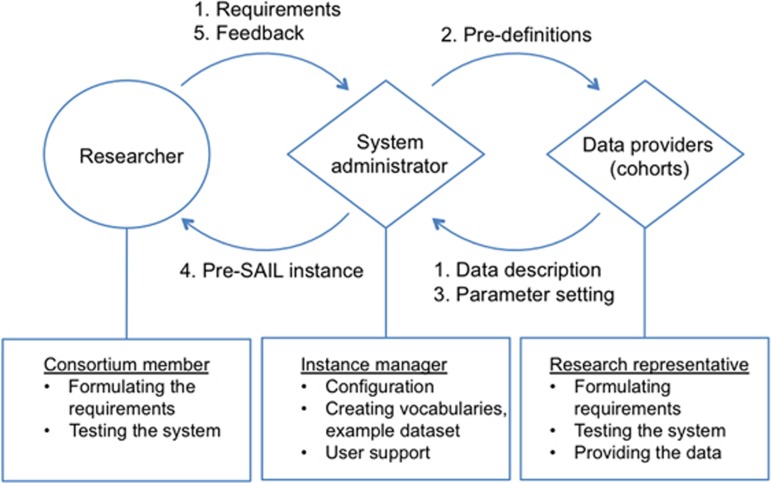
Workflow and responsibilities for the iterative harmonisation process in the SAIL method, involving multiple curation teams and facilitated by a web-based application. (1) Providing a description of the available data from individual cohorts and the requirements from the researcher. (2) Based on this data, the system administrator creates pre-definitions of a possible parameter setting. (3) Testing and verifying the parameter setting by data provider. (4) Offering a pilot instance to the consortium user for checking and verifying the system or for getting any feedback for further alterations to the configuration. (5) Feedback is provided by the researcher and the entire process is iterated.

Based on the experiences obtained in various projects and consortia, we present in Figure 2 a typical harmonisation workflow for the SAIL method facilitated by a web-based system. The process involves researchers, system administrators and local data managers at sample collections. Definitions of variables are formulated by leading researchers, then translated into a system configuration and revised by data providers (data managers at biobanks). On receipt of feedback from cohorts, terms are revised and the next version is released.

## Results

We have applied the SAIL methodology in three projects (Table 2): (1) within the ENGAGE consortium that pioneered the method; (2) for linking Swedish national biobanks with clinical registry data at the Karolinska Institutet; and (3) for generating information about sample availability in the SUMMIT project. It should be noted that all three applications consistently used the same methodology to achieve harmonised and interlinked data.

### Case study 1: sample availability in the ENGAGE consortium

The ENGAGE consortium was established in 2008, with the main objective of sharing and analysing data from a number of already established cohorts comprising more than 80 000 GWAS scans, and DNA and serum/plasma samples from over 600 000 individuals^16^ in 39 cohorts distributed over 18 partner organisations. The SAIL method was applied within the ENGAGE consortium in the following steps:

- Data modelling and design.
- Mapping and data collection.

#### *Data modelling and design*

*Formulation of use cases.* The main driver behind the harmonisation work in ENGAGE was the need for fast quantification of the number of samples that were potentially available for genome-wide meta-analysis studies across multiple cohorts. A set of use cases was identified through multiple discussions with potential users (statisticians and epidemiologists), for instance:

- For how many DNA samples in each cohort are there type 2 diabetes status records, as well as fasting glucose concentration and BMI?
- Which covariates can be used during the analysis?
- How many samples would be available if the study was limited to individuals younger than 45 years old?

Requirements for the data submission format and interactive interface were also collected during these meetings and through analysis of the harmonisation and mapping workflow.

*Sample data.* Sample data that were provided by participating biobanks came with Supplementary Information on in-house descriptions of data types and data models. Data types were assessed and decisions were made on their suitability.

*Semantic information.* For the organisation of indexing terms to be used in SAIL and their relationships, a number of existing vocabularies and resources for creation, storage and mapping of ontologies were reviewed: GO,^14^ OBO,^17^ EFO,^18^ phenX^7^ and dbGAP.^19^ Several initiatives and projects based on semantic web technologies providing solutions for tagging objects with concepts and inter-relating the concepts, such as conceptWiki, and structured semantic search tools, such as ViziQuer,^20^ were surveyed. First, a draft structure for capturing information about variables in SAIL was proposed. Next, it was completed and refined iteratively over several rounds of loading data, testing and discussing with the users.

#### *Mapping and data collection*

The first prototype of SAIL was test run on a cumulative index of samples from 10 collections within the ENGAGE consortium. The index was based on 61 variables, which were suggested by data analysts from the University of Oxford and Institute for Molecular Medicine Finland (FIMM) working on the identification of genetic markers for diseases including type 2 diabetes and cardiovascular disease. Selected VOIs were grouped in a metabolic syndrome (MetS) vocabulary (Supplementary S1). The initial format for the description of terms (name, definition, unit, time point, etc) was suggested by epidemiologists and subsequently cross-checked against the standard format proposed by Data Schema and Harmonization Platform for Epidemiological Research (DataSHaPER)^6^, the major international initiative for the best practices in biospecimen data harmonisation. On finalisation of the harmonised MetS vocabulary, the local data managers at each collection:

- Mapped local sample descriptions (variables) to MetS,
- Extracted sample data from the biobank database for those samples that were relevant to at least some of the variables in MetS,
- Replaced the values with 1 and missing values with 0 or left them blank in the extracted matrix and
- Sent the availability matrix to the SAIL development team.

Collaborating cohorts that were not part of the ENGAGE consortium submitted the second batch of data. Data were either provided in the MetS vocabulary or, in case of a different clinical scope, in other vocabularies. In the latter case, related variables from individual vocabularies were linked in SAIL. A list of data contributors is available in Table 3.

### Case study 2: using SAIL to link biobanks with clinical data

Clinical health registries record information about patients in health care, with the main objective to be able to follow up on the quality of health care and also provide a gold mine of data for research.^21, 22^ The clinical data in national health registries are often highly sensitive and administered by physicians. In a previous study, a federated architecture of clinical registries was suggested and implemented in Sweden.^23^ Such a system, however, assumed adoption of exactly the same database schema by all interconnected resources (ie, biobanks in this case), which proved to be costly and often not feasible due to differences in the underlying medical protocols and standard operating procedures.

We applied the SAIL method to a biobank at the Karolinska Institutet, Sweden, containing biospecimens in the form of DNA, serum and blood from patients. We integrated this availability data with a selected subset of the Swedish national prostate cancer quality registry comprising information on diagnosis, treatment and follow-up. A set of use cases was defined to be:

- For how many prostate cancer patients older than 60 years with a Gleason score above 6 do we have DNA stored in the biobank?
- For the patients with regional lymph node metastasis present, how many have answered a questionnaire and have blood plasma available in the biobank?
- For patients diagnosed with prostate cancer between the year 1990 and 2010 with a PSA value above 8, how many have DNA or blood plasma stored in the biobank?

The information originated from different resources and was linked using the patient's Swedish personal number and a custom vocabulary that was developed (Supplementary S2). A demo version of the SAIL system of the same structure and populated with simulated values is available from sail.simbioms.org/bbqr.

### Case study 3: sample availability within the SUMMIT consortium

SUMMIT (http://imi-summit.eu) is a pan-European research consortium that works on the systematic identification of genetic risk factors for chronic diabetic complications. A collection of patient samples from a variety of cohorts was analysed by high-throughput techniques, for example, genotyping, and both patient samples and genotypes were harnessed for biomarker discovery. The data provided by consortium participants were either the actual measured data values or values indicating availability (if a value exists for a given phenotype and individual then 1, otherwise 0). Users are thus able to query SAIL to obtain estimates of how many individuals fulfill certain criteria, for example, to select the most informative individuals within SUMMIT for GWAS genotyping and omics analysis. Examples of use cases include:

- For how many T2D patients older than 35 years with myocardial infarction would there be DNA samples available?
- How many non-diabetic individuals of female gender, age 18–45 years, would have pre-existing GWAS data?
- For how many T2D patients is there data available on the status of proliferative retinopathy or maculopathy?

In the first stage of applying the SAIL method, it was necessary to estimate informative and available data that users should be able to query. This process required fluent interaction between the data manager, system administrator and researcher (consortium user). Based on the collected information, a pre-configuration set was developed and the vocabulary was sent for revision to a test user who helped finalise the variable definitions (Supplementary S3).

## Discussion

The lack of a comprehensive methodology for linking information across data providers reduces the ability of researchers to combine data from disparate sources. The SAIL method is the first to formalise and test a methodological framework for interlinking informative records from a variety of biomedical archives: biobanks, health registries and various studies. Originally developed to collect sample availability information for the ENGAGE consortium, the SAIL method has since been applied and evaluated in several other projects. The three applications of the SAIL method described herein demonstrate the method's generality: the ENGAGE case showed how SAIL is capable of interlinking hundreds of thousands of samples in a public SAIL instance, whereas the SUMMIT instance showed the feasibility of the method for a completely different endpoint that had more restrictive security demands and was set up within a secure network. The application in which SAIL was used to integrate biobank data with a clinical cancer registry in Sweden illustrates the potential for the SAIL method to go beyond biobank data integration and opens up opportunities for new types of translational studies, such as including genotype data when estimating treatment success. Indexing availability data without collecting the actual data values has in this case been of great importance to gain acceptance among data providers. As health registries and biobanks traditionally are geographically as well as operationally separated, adoption of SAIL can enhance biobank research by linking data from diverse sources. SAIL has no built-in restriction on the nature of the data stored, and in this case the data items that usually describe biospecimen samples in the context of biobanks are represented by data describing patients.

It should be noted that the SAIL method does not resolve privacy issues but rather permits researchers to sidestep privacy-related obstacles and procedures in the planning phase, which for large projects may represent a significant time-saving due to unnecessary data access requests for biobanks that do not contain relevant data. In the later phase of the project, when the real data are to be exchanged and the application for the data access is to be filed, the procedural constraints involved in full data access applications are unavoidable. The amount of work the researchers put towards an appropriate project plan should not be underestimated. The SAIL method simplifies obtaining accurate availability information, which may significantly reduce time and effort required for filing and processing the required data access requests. The three case studies presented here show the method's applicability in both public and private settings with restricted access, and the vocabularies and mappings developed for these projects can also be used in future studies.

The SAIL method requires all participating collections to provide specific information about each variable: what is measured, how the measurement was performed, who performed the measurement and what were the accompanying factors, for example, ‘fasting'. This level of detail allows comparison of what was measured in research projects and enables decisions on whether variables can be considered to be equivalent for a certain analysis or could potentially be brought to an equivalent status by some transformation (for example, (*N* smoked cigarettes per day) × 7=*N* smoked cigarettes per week). When variables are fully equivalent, they can be related to each other as ‘full synonyms'. If one is a subcase of another, they can be marked as parent and child. If variables are related, but are neither of the above, they are flagged as a partial match. Relating variables recorded at the time of collection to the variables requested by researchers conducting a cross-cohort project as accurately as possible and preserving such mappings has two significant implications for everyday work of researchers at biobanks and in genomics centres. First, it helps to annotate samples in a harmonised/consistent manner, which had originally been described differently, thus making data available for federated queries. Second, it helps to avoid re-mapping the same variables between collections repetitively, for example, for various omics consortia.

The complementing SAIL software was the first system to provide online searching for sample availability in meta-analysis of human cohorts. It is also the only resource to provide cross-biobank sample availability for the cohorts that contributed to the ENGAGE consortium. We do not regard the SAIL software as the only viable software solution for this method; in fact, we would like to encourage the research community to develop other suitable software solutions. A major motivation for an online availability system is the increased sample visibility it gives, which could result in greater opportunities to highlight the scientific value of biobank content, for example, identifying samples that have been used in many studies or those that have rare phenotypes or data associated with them.

The more collaborations a biobank establishes, the higher the volume of requests for harmonisation. The processing of requests involves local epidemiology and informatics expertise, and each request is slightly different, even when requests concern the same variables. The SAIL method is capable of indexing the information about underlying medical protocols, questionnaires and contexts of meta-projects. In addition, the method captures the details of mapping between variables and thus allows tracking and re-using the same mappings in future studies.

In addition to harmonised variables, collaborative studies also require understanding the designs used to collect the data in the individual studies. Accounting for the different study designs in the analysis is especially important if the objective is to estimate population statistics, absolute risks or causal effects. As the commonly used design names, such as cohort study, case–control study or case–cohort study, are ambiguous by themselves^24, 25^, the verbal definitions can be complemented with more systematic ways to describe the design.^26^ This remains as a potential direction of future development for the SAIL method.

The main limitation of the approach we have presented, not uncommon among harmonisation solutions in biomedical informatics, is the lack of an interface with other approaches. When it comes to data management, many tools, approaches and practices solve one particular problem well, whereas for researchers it is vital to have a comprehensive solution for a complete range of data annotation and processing problems. For instance, the DataSHaPER platform and SAIL have partial overlap in functionality and methodology for harmonisation of data schemas, but some aspects of the process are different: central curation *vs* distributed curation, that is, how much the data schema is governed by curators working within the platform and how much is in the hands of submitters. It is crucial for the data providers to be fully aware which scenarios of metadata and availability data submission are supported by which platform, in order to make informed choices of harmonisation tools. Interoperability between stand-alone data harmonisation platforms and frameworks is yet to be developed and is being targeted by large consortia such as ELIXIR (http://www.elixir-europe.org) and BBMRI-ERIC (http://bbmri-eric.eu). We also acknowledge that generic evaluation mechanisms for interoperability projects and methodologies are urgently needed, but this topic is not directly addressed in this study. Our future work will aim to address this, using action design research and building on best practices from business sciences for this purpose.

Much of the success of SAIL depends on harnessing ongoing community efforts to build biomedical ontologies and vocabularies. Annotation with community-wide ontologies allows integrated searches to be performed across disparate data sources and maximises visibility for both primary data and research results. Through its formalism, the SAIL method empowers consortia, collaborative initiatives and individual biobanks to interlink existing and future data across various biomedical research and healthcare digital collections. The features of SAIL thereby greatly enhance the efficiency of translational and multi-disciplinary research efforts.

## Figures and Tables

**Table 1:** Terminology used in this manuscript

Specimen	An individual portion of human, animal, plant, mineral and so on, materials used for scientific research project
Biospecimen	An individual portion of a substance of biological origin, for example, tissue sample, blood sample, saliva sample and so on, derived from a single participant at a specific time and intended to be used for scientific research project, which in the context of this study is stored in a biobank
Sample	A synonym for ‘biospecimen', also called ‘biosample', meaning, for example, a blood sample, tissue sample, urine sample and so on A number of biospecimens selected for a particular scientific research project intended to be representative of a given population. For example, an experimental sample might contain 200 cancerous tissue biospecimen samples from various individuals across Europe or 1000 biospecimens of blood taken from various individuals within the United Kingdom
Biomedical data archive or data bank	A storage and retrieval facility or service for biological and medical data. All data archives have three primary functions: the collection, storage and preservation of data
Phenotypic variables	A characteristic that varies across a population of interest, for example, height, weight, eye colour, blood pressure and the presence or the absence of various clinical conditions such as diabetes
VOI	A phenotypic or genotypic variable that is relevant for a particular research project. A selection of such variables is referred to as the VOIs for the research project
HV	A single unified vocabulary that has been compiled from several individual vocabulary sources. Where there is partial overlap in the meaning of terms from separate vocabularies but with different exact labels used, synonyms from each of the underlying vocabularies are preserved in the resulting HV
Metadata	Information about, or description of, data. The metadata describing a biospecimen sample collection might include, for example, the number of specimens stored in the collection and summary statistics about the population from which the specimens were collected
CV	A list of words and phrases intended for use to mark up or index data, selected such that each unit in the vocabulary is unique and unambiguous within the overall vocabulary and thereby the use of controlled vocabularies ensure consistency in annotation
GWAS	Examines genetic variants, such as SNPs, across the genome in various individuals to see whether any variant is associated with a phenotype, for example, a disease such as diabetes

Abbreviatons: CV, controlled vocabulary; GWAS, genome-wide association study; HV, harmonised vocabulary; SNP, single-nucleotide polymorphisms; VOI, variables of interest.

These definitions have been synthesised and modified from various sources and discussed among the authors, in order to achieve consistency across the manuscript. Many of these terms are used in different ways in different contexts.

**Table 2:** Summary of three applications where the SAIL method was applied

	*ENGAGE*	*Karolinska Institutet*	*SUMMIT*
Number of linked individuals/samples	184 000	1000	30 494
Number of linked collections	15	2 (1 Biobank+1 health registry)	15
Number of harmonised variables	92	13	43
Availability software used	SAIL	SAIL	SAIL
Key purpose	Sharing and analysing the data from 39 cohorts among 18 consortium partners	Identify subsets in health registry for which there are biobank data available	Assistance in design of GWAS meta-studies for complications in diabetic patients
Vocabulary	MetS (Supplementary S1)	bbqr (Supplementary S2)	Summit (Supplementary S3)
Web address	Public: sail.simbioms.org	Public (simulated data): sail.simbioms.org/bbqr Private: restricted access user: sailuser, pwd: karolinska	Public (simulated data): sail.simbioms.org/summit Private: restricted access

Abbreviations: ENGAGE, European Network for Genetic and Genomic Epidemiology; GWAS, genome-wide association study; MetS, metabolic syndrome; SAIL, sample availability; SUMMIT, surrogate markers for micro- and macro-vascular hard endpoints for innovative diabetes tools.

For the Karolinska Institutet and SUMMIT projects there is a public instance of the SAIL software with simulated data, whereas the private instance with real data has restricted access.

**Table 3:** Data contributors and institutions participated in mapping activities and data submission for the ENGAGE application harmonised with the SAIL method

*Collection(s)*	*Representing organisation*
MolOBB	Oxford Centre for Diabetes, Endocrinology and Metabolism, Churchill Hospital, Old Road, Oxford OX3 7LJ, UK
NFBC66, Genmets case, Genmets control	FIMM, THL and University of Helsinki, Biomedicum Helsinki 2U, 00014 Helsinki, Finland
UK-twin	King's College London, UK
ERF	Department of Epidemiology and Biostatistics, Erasmus University Medical School, 3000 DR Rotterdam, The Netherlands
DGI	Lund University Diabetes Centre, Malmö, Sweden
EGCUT	The Estonian Genome Center of University of Tartu
KORAF3, KORAF4	Helmholtz Zentrum München German Research Center for Environmental Health (GmbH)
STR	Karolinska Institutet (Karolinska)
	
*Additonal submissions*	
HUNT1, HUNT2, HUNT3	HUNT Research Centre, Norwegian University of Science and Technology (NTNU), Trondheim, Norway
Latvian Genome Data Base (LGDB)	Genome Centre, Latvian Biomedical Reserch and Study centre, Ratsupites 1, Riga LV-1067, Latvia

Abbreviations: ENGAGE, European Network for Genetic and Genomic Epidemiology; SAIL, sample availability.
