# Supplementary material for: Harmonising and linking biomedical and clinical data across disparate data archives to enable integrative cross-biobank research
Source: Eur J Hum Genet. 2015 Aug 26;24(4):521–8. doi: 10.1038/ejhg.2015.165 (PMC4929882; doi:10.1038/ejhg.2015.165)
Supplement: Supplementary Information S3 [file ejhg2015165x3.pdf]

SUMMIT

surrogate markers for
micro- and macro-vascular
hard endpoints for
innovative diabetes tools

SAIL documentation

for use within

SUMMIT

Variable definitions for upload of individual level information on diabetic complications into Sample avAILabilty system (SAIL)

17 March 2011

Introduction

SAIL is a web-based application for searching, browsing and annotating biological sample collections. By providing individual-level information on the availability of specific variables or phenotypes resource integration can be facilitated. The provided data can be either the actual measurement data or just indicating if a value exists for a given phenotype and individual. When data is available, users can query SAIL in order to get estimates of how many individuals that fulfil certain criteria. For example, SAIL can help to select the most informative individuals within SUMMIT to choose for GWAS genotyping. For more information on SAIL, please visit the first instance of SAIL at EBI (www.ebi.ac.uk/Tools/sail/) where a tutorial is available.

We ask all SUMMIT partners to upload information on all their cohorts with variables encoded as specified in this document. When in place, we expect SAIL to be a very useful tool for several SUMMIT work packages.

This document includes a table with all variables specified. Following the table is some extra information on how to encode each variable.

Please contact Michael Hillström, Michael.Hillstrom@med.lu.se, when ready to upload your cohort information to decide upon most convenient data transfer option.

General questions regarding SAIL can be sent by email to either:
Michael Hillström, Michael.hillstrom@med.lu.se
Claes Ladenvall, claes.ladenvall@med.lu.se
Mark McCarthy, mark.mccarthy@drl.ox.ac.uk

Terminology

The following terminology is used throughout the whole document:

Case An individual with diabetes and with the complication of interest.
Control An individual with diabetes but without the complication of interest.
Other An individual where information is available, but who does not fulfil the case or control criteria
Unknown Information is not available

Table 1. Variable definitions

Variable number	Variable name	Variable description	Datatype	Value	Value description
1	ID	Unique identifier for individuals	Text		
2	COHORT_NAME	Cohort name	Text		
3	GENDER	Gender	Alternative	0	Male
				1	Female
				-9	Unknown
4	DIABETESTYPE	Diabetestype	Alternative	0	Non-diabetic
				1	T1D
				2	T2D
				3	Diabetes confirmed, but other than T1D and T2D.
				4	Unknown diabetestype
5	AGE_DIAB_DIAG	Age in years at diabetes diagnosis	Numeric		
6	DNA	DNA available	Alternative	0	No
				1	DNA
				2	Blood
				-9	Unknown
7	WGA_DNA	Is the DNA Whole Genome Amplified	Alternative	0	Native
				1	WGA
				-9	Unknown
8	GWAS	GWAS performed	Alternative	0	No
				1	Yes
				2	Other (i.e metabochip)
				-9	Unknown
9	CHD1	Fatal or non-fatal myocardial infarction	Alternative	0	No
				1	Yes
				-9	Unknown
10	CHD2	Unstable angina	Alternative	0	No
				1	Yes
				-9	Unknown
11	CHD3	Interventions	Alternative	0	No
				1	Yes
				-9	Unknown
12	STROKE	Fatal or non-fatal ischemic stroke	Alternative	0	No
				1	Yes
				2	Other (i.e hemmorrhagic)
				-9	Unknown
13	AGE_CHD1	Age in years at CHD1 case diagnosis	Numeric		
14	AGE_CHD2	Age in years at CHD2 case diagnosis	Numeric		
15	AGE_CHD3	Age in years at CHD3 case diagnosis	Numeric		
16	AGE_STROKE	Age in years at stroke case diagnosis	Numeric		

17	AGE_CVD_CHECK	Age in years at last evaluation of CVD status	Numeric		
18	DN	Diabetic nephropathy	Alternative	0	Control
				1	Microalbuminuria
				2	High microalbuminuria
				3	Macroalbuminuria
				4	End stage renal disease
				5	Other, does not fulfil case or control criteria
				-9	Unknown
19	AGE_DN	Age in years at nephropathy case diagnosis	Numeric		
20	AGE_DN_CHECK	Age in years at last evaluation of DN status	Numeric		
21	DR1	Mild-moderate non-proliferative retinopathy	Alternative	0	No
				1	Yes
				2	Other (does not fulfil case or control criteria)
				-9	Unknown
22	DR2	Severe non-proliferative retinopathy	Alternative	0	No
				1	Yes
				2	Other (does not fulfil case or control criteria)
				-9	Unknown
23	DR3	Proliferative retinopathy (requires at least 45 degrees fundus photograph)	Alternative	0	No
				1	Yes
				2	Other (does not fulfil case or control criteria)
				-9	Unknown
24	DR4	Proliferative retinopathy (requires panretinal laser therapy)	Alternative	0	No
				1	Yes
				2	Other (does not fulfil case or control criteria)
				-9	Unknown
25	DRM1	Maculopathy (based upon at least 30 degrees fundus photograph)	Alternative	0	No
				1	Yes
				2	Other (does not fulfil case or control criteria)
				-9	Unknown
26	DRM2	Maculopathy (based upon central laser therapy)	Alternative	0	No
				1	Yes
				2	Other (does not fulfil case or control criteria)
				-9	Unknown

27	AGE_DR1	Age in years at DR1 case diagnosis	Numeric		
28	AGE_DR2	Age in years at DR2 case diagnosis	Numeric		
29	AGE_DR3	Age in years at DR3 case diagnosis	Numeric		
30	AGE_DR4	Age in years at DR4 case diagnosis	Numeric		
31	AGE_DRM1	Age in years at DRM1 case diagnosis	Numeric		
32	AGE_DRM2	Age in years at DRM2 case diagnosis	Numeric		
33	AGE_DR_CHECK	Age in years at last evaluation of DR status	Numeric		
34	LEAD	Lower extremity arterial disease	Alternative	0	No
				1	Yes
				2	Other (does not fulfil case or control criteria)
				-9	Unknown
35	AGE_LEAD	Age in years at LEAD case diagnosis	Numeric		
36	AGE_LEAD_CHECK	Age in years at last evaluation of LEAD status	Numeric		

VARIABLE SPECIFICATIONS

1. ID

Unique identifier for individuals. These keys are needed to let users identify the DNA samples that correspond to samples of interest defined through the use of SAIL. Please use a three-letter-code as a prefix to the identifier to ensure that unique keys are used. Examples of three-letter-codes are:
ULU=Lund University
UDU=University of Dundee
UEX=University of Exeter

2. COHORT_NAME

The name of the cohort.

3. GENDER

The individual's gender.

4. DIABETESTYPE

Diabetes is defined on the basis of contemporary or historical evidence of hyperglycaemia (according to WHO 1998 criteria; fasting plasma glucose >= 7.0 mmol/l or 2-h plasma glucose >= 11.1mmol/l, or both) or by current medication with insulin, sulphonylureas, metformin or other antidiabetic drugs.

Value	Value description	Comment
0	Non-diabetic	Individual that hasn't been diagnosed with diabetes.
1	T1D	To define T1D, individuals should have been diagnosed before the age of 35 and have required insulin treatment from diabetes onset.
2	T2D	To define T2D, individuals should have been diagnosed after the age of 30 and clinical, immunological (no GAD or other islet cell antibodies) and genetic tests (not MODY) (where these tests have been performed) should be consistent with the diagnosis.
3	Diabetes confirmed, but other than T1D and T2D	
4	Diabetes status unknown	

5. AGE_DIAB_DIAG

Age in years, at time of diabetes diagnosis.

6. DNA

Indicate if sufficient DNA (approx. 750 ng) is available for genotyping / GWAS, if it has to be extracted *de novo* from available blood/buffy coats, or if no DNA is available.

7. WGA_DNA

Indicate if the DNA is whole genome amplified (WGA) or native.

8. GWAS

This indicates if a genome wide chip has been run. Whenever only metabochip or other medium scale chips have been used, please indicate this using value=2.

9. CHD1

Definite or possible fatal or non-fatal myocardial infarction. Please note that we are interested in diabetic complications. Thus, we are primarily interested in information on individuals that have developed CHD1 after diabetes onset. However, combining the information in the AGE_CHD1 and AGE_DIAB_DIAG variables will allow us to determine the difference in time between the diagnoses of the 2 events. This also applies to a number of other variables below.

10. CHD2

Unstable angina. Please note the comment on the CHD1 variable.

11. CHD3

Any coronary intervention (i.e coronary artery bypass graft or other coronary revascularization procedure). Please note the comment on the CHD1 variable.

12. STROKE

Fatal or non-fatal ischaemic stroke. Stroke is defined as rapidly developed clinical signs of focal or global disturbance of cerebral function lasting more than 24 hours (unless interrupted by surgery or death), with no apparent cause other than a vascular origin. Please note the comment on the CHD1 variable.

It does NOT include:
Subarachnoid haemorrhage
Stroke known to be due to intracerebral haemorrhage
Or transient cerebral ischaemia (TIA) i.e. focal deficits lasting < 24 hours without imaging confirmation of a stroke
Or stroke events in cases of blood disease (e.g. leukaemia, polycythaemia vera), brain tumour or brain metastases.
Or secondary stroke caused by trauma
Or prior carotid artery surgery for atheromatous occlusion

The “Other” category (value 2) should be used for any individual who has suffered a stroke that does not qualify as ischaemic stroke.

13. AGE_CHD1

Age in years, at time of first fatal or non-fatal myocardial infarction. Should only be reported for CHD1 cases.

14. AGE_CHD2

Age in years, at time of diagnosis of unstable angina. Should only be reported for CHD2 cases.

15. AGE_CHD3

Age in years, at time of first coronary intervention. Should only be reported for CHD3 cases.

16. AGE_STROKE

Age in years, at time of first fatal or non-fatal ischaemic stroke. Should only be reported for STROKE cases.

17. AGE_CVD_CHECK

Age in years, at time of last evaluation of cardiovascular events (CHD1, CHD2, CHD3 and stroke). This variable will be used to calculate diabetes duration in CHD1, CHD2, CHD3 and STROKE controls. Should be filled in for all individuals that are not Unknown (-9) for these variables.

18. DN

Diabetic nephropathy is subdivided into microalbuminuria, high microalbuminuria, macroalbuminuria and endstage renal disease according to the following definitions:

**Value**	**Value description**	**Comment**
0	Control	Normoalbuminuria (AER <20 µg/min or <30 mg/24 hr or ACR <2.5 for men and <3.5 for women) at all visits.
1	Microalbuminuria	At least 2 out of 3 consecutive measurements with AER ≥20, <100 µg/min or ≥30, <150 mg/24 hr or ACR ≥2.5, <12.5 for men and ≥3.5, <17.5 for women.
2	High microalbuminuria	At least one measurement with AER ≥100, <200 µg/min or ≥150, <300 mg/24 hr or ACR ≥12.5, <25 for men and ≥17.5, <35 for women.
3	Macroalbuminuria	At least one measurement with AER ≥200 µg/min or ≥300mg/24 hr or ACR ≥25 for men and ≥35 for women
4	End stage renal disease	Defined as eGFR ≤15 ml/min or dialysis or kidney transplantation.
5	Other, does not fulfil case or control criteria	
-9	Unknown	

Note: An individual should only belong to the most severe group that the individual can qualify for.

Albuminuria is classified based on timed overnight urinary albumin excretion rate (AER, µg/min or mg/24 h) or an albumin-creatinine ratio (ACR, mg/mmol) in a first morning urine sample.
The renal function (eGFR) is estimated using the MDRD-4 formula:

For creatinine in mg/dL:

$$\text{eGFR} = 186 \times \text{Serum Creatinine}^{-1.154} \times \text{Age}^{-0.203} \times [1.210\ if\ Black] \times [0.742\ if\ Female]$$

For creatinine in µmol/L:

$$\text{eGFR} = 32788 \times \text{Serum Creatinine}^{-1.154} \times \text{Age}^{-0.203} \times [1.210\ if\ Black] \times [0.742\ if\ Female]$$

Creatinine levels in µmol/L can be converted to mg/dL by dividing them by 88.4. The 32788 number above is equal to $186 \times 88.4^{1.154}$.

These MDRD equations are to be used only if the laboratory has NOT calibrated its serum creatinine measurements to isotope dilution mass spectroscopy (IDMS). When IDMS-calibrated serum creatinine is used (which is about 6% lower), the above equations should be multiplied by 175/186 or by 0.94086.

Stages of renal function are defined as follows (KDOQI)

Stage I	eGFR	> 90 ml/min	Normal
Stage II	eGFR	60-90 ml/min	Mildly reduced
Stage III	eGFR	30-60 ml/min	Moderately reduced
Stage IV	eGFR	15-30 ml/min,	Severely reduced
Stage V	eGFR	<15 ml/min	End-stage renal disease

Chronic kidney disease is considered present when eGFR is <60 ml/min (stages III-V).

19. AGE_DN

Age in years at diagnosis of nephropathy as specified in variable DN. Should only be reported for DN cases and refer to the age of diagnosis of the most severe class of DN suffered.

20. AGE_DN_CHECK

Age in years, at time of last evaluation of diabetic nephropathy. This variable will be used to calculate diabetes duration in DN controls. In cases, it may be used to check that individuals with less severe DN have not progressed to more severe DN. Should be filled in for all individuals that are not Unknown (-9) for DN.

21. DR1

Mild-moderate non-proliferative retinopathy, (requires at least 45° fundus photograph). Please report the status of the “worse” eye.

Diagnosis of diabetic retinopathy can be based upon either information on fundus photography, ophthalmoscopy or laser treatment for diabetic retinopathy.
Fundus photographs cover varying parts of the retina, usually 30 degrees, 45 degrees or 50-60 degrees. To be informative for definition of proliferative retinopathy we would require at least 45-degree coverage, for maculopathy 30 degrees will be sufficient.
Laser therapy: information on laser treatment is based upon either fundus photographs, ophthalmoscopy or medical records.

22. DR2

Severe non-proliferative retinopathy, includes IRMA (intraretinal microvascular abnormalities) - requires at least 45° fundus photograph. Please report the status of the “worse” eye.

For more information on the diagnosis of diabetic retinopathy, please see DR1 above.

23. DR3
Proliferative retinopathy - requires at least 45° fundus photograph. Please report the status of the "worse" eye.

For more information on the diagnosis of diabetic retinopathy, please see DR1 above.

24. DR4
Proliferative retinopathy based upon pan-retinal laser therapy. Please report the status of the "worse" eye.

For more information on the diagnosis of diabetic retinopathy, please see DR1 above.

25. DRM1
Maculopathy based upon at least 30° fundus photograph. Please report the status of the "worse" eye.

26. DRM2
Maculopathy based upon central laser therapy. Please report the status of the "worse" eye.

27. AGE_DR1
Age in years at DR1 diagnosis.

28. AGE_DR2
Age in years at DR2 diagnosis.

29. AGE_DR3
Age in years at DR3 diagnosis.

30. AGE_DR4
Age in years at DR4 diagnosis.

31. AGE_DRM1
Age in years at DRM1 diagnosis.

32. AGE_DRM2
Age in years at DRM2 diagnosis.

33. AGE_DR_CHECK
Age in years, at time of last evaluation of retinopathy (or maculopathy) (DR1, DR2, DR3, DR4, DRM1 and DRM2). This variable will be used to calculate diabetes duration in DR1, DR2, DR3, DR4, DRM1 and DRM2 controls. Should be filled in for all individuals that are not Unknown (-9) for these variables.

34. LEAD

Prior corrective surgery, angioplasty, or any amputation of the extremities.

35. AGE_LEAD

Age in years at first LEAD diagnosis. Should only be reported for LEAD cases.

36. AGE_LEAD_CHECK

Age in years, at time of last evaluation of LEAD. This variable will be used to calculate diabetes duration in LEAD controls. Should be filled in for all individuals that are not Unknown (-9) for LEAD.
