# Supplementary material for: Harmonising and linking biomedical and clinical data across disparate data archives to enable integrative cross-biobank research
Source: Eur J Hum Genet. 2015 Aug 26;24(4):521–8. doi: 10.1038/ejhg.2015.165 (PMC4929882; doi:10.1038/ejhg.2015.165)
Supplement: Supplementary Information S4 [file ejhg2015165x4.pdf]

S4: SAIL METHOD SUPPLEMENT

In the SAIL method, each vocabulary file consist of two sections: the common tags section (optional), where tags can be used to classify all parameters if defined in the beginning of the file or just for a single parameter if defined in the parameter section and the parameter section, which is divided into the main parameter section and a variable definition section, see Figure F1 for the format definition and Figure F2 for an example vocabulary file.

	Definition	Value	Value	
Common Tag	Tag	String	String	
Paramater section	Code	String [a-zA-Z0-9:]		
	Name	String		
	Description	String [\n means new line if multiline Description is needed]		
	Tag	String	String	
	Inherit	String		
	Relation	String	String	String
Variable definition section	Variable	String		
	Type	String [STRING,INTEGER,REAL,BOOLEAN,ENUM]		
	Description	String		
	Variant	String		
	Predefined	Boolean [true,false]		

Figure F1 Metadata import format in the SAIL method. The highlighted sections are the format specifications, *i.e.* a tab-delimited text file. Each underlined definition is mandatory.

Listing F2 An example metadata file in SAIL format.

Example:

```
Tag	Vocabulary	MetS
Tag	Knowledge	Domain

Code		MetS:BP
Name		Blood pressure
Description	Blood pressure
Tag		Organ	Body
Relation	P3G:BP		Standard relations	Full synonym
Variable	Systolic
Type		INTEGER
Description	Systolic blood pressure
Variable	Diastolic
Type		INTEGER
Description	Diastolic blood pressure

Code		MetS:GLU
Name		Glucose
Description	Glucose, mMol/L
Tag		Organ	Blood
Relation	P3G:GLU		Standard relations	Synonym
Variable	Concentration
Type		REAL

Code		MetS:GLUTM
Name		Glucose with timing
Description	Glucose with timing, mMol/L
Tag		Organ	Blood
Inherit		MetS:GLU
Qualifier	Timing
Variant		Fasting		1
Variant		Non-fasting	2
Predefined	true
```

The *data and availability information* format is equally suitable for sample data collection or for sample availability information, as illustrated in the example below. In the first case, the matrix of Sample IDs (rows) vs. Harmonised Terms (columns) is filled with actual parameter values (see Sample 1, MetS:BP and MetS:GLUTM.concentration). In the second case, i.e. when only availability data can be collected, the matrix contains "0" and "1" for "value is not recorded" and "value is available" for each sample-parameter pair. Other types of coding (@ - available, null – not on record) can be applied too. Type of coding is to be agreed upon beforehand and between the data providers and the administrators of the availability system.

Sample.ID	MetS:BP	MetS:GLUTM.concentration	MetS:GLUTM.Timing
**sample1**	80	4	Fasting
**sample2**	@	@	1
**sample3**	3	@	

Furthermore, the visibility of samples from a certain collection can be increased by additional classification of variables that are used to characterize the samples: by assigning a variable to a vocabulary, a research project or a canonical phenotype. An illustration on the user interface for query construction is available in Figure F3.

Main limitations of SAIL method, similarly to other approaches in bioinformatics – lack of interface with other approaches. In spite of data import and export being in XML and rdf, nonetheless, from a researchers perspective, SAIL is stand-alone platform. Many tools, approaches and practices when it comes to data management solve one particular problem well, while for researchers it is vital to have a comprehensive solution for a complete range of data annotation and processing problems. Among underlying reasons for lack of interoperability and compatibility between various platforms for data handling is lack of interoperability between existing data management services. ELIXIR-ERIC aims to bridge the gap between the services by providing scientific community with best practices how to build services, how to annotate Life Sciences and biomedical data in a such a way that several solutions could be combined as if a single platform. In line with ELIXIR-ERIC, BBMRI-ERIC tackling specifically the interoperability for biobanks. We hope that in future, SAIL and similar approaches will have the dedicated IT and data curation means, already from the stage of design, that will allow for creation of seamless interfaces and integration accross several solutions.

Implementation

The SAIL software is implemented as a client-server application. The client part is developed with Google Web Toolkit (GWT) and the Ext-JS widget library, and runs in a regular web browser. The technical SAIL description has additional information on the architecture.

To install a SAIL instance, a server running a servlet container (such as Tomcat, JBoss or similar) and a local database such as MySQL or PostgreSQL is required. SAIL itself is distributed as a Web Archive (WAR), and can be installed by simply

placing it within the specified folder in the servlet container. A database schema for building the structure of the MySQL database is also provided in the download. After this, some configuration settings need to be adjusted to suit the local environment. The installation guide of SAIL covers all aspects of the installation.

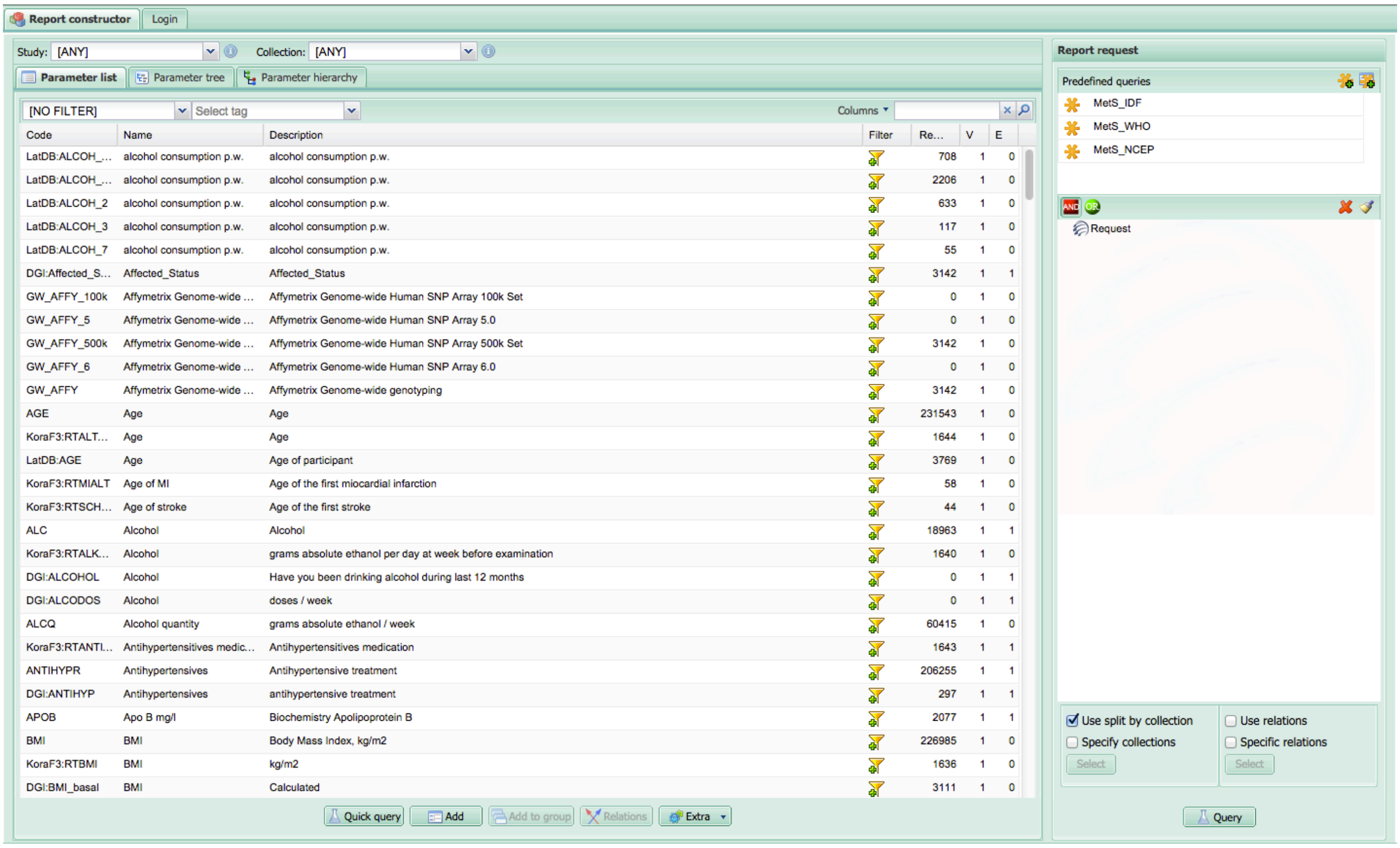

Figure F3 The SAIL report constructor screen view consists of a list of parameters that can be filtered for free text, or for tags associated to any classifier. Additional filters can restrict this list to parameters that have samples associated to them within a specific research project or specific cohort. The right hand view of the window is the report request, formed by parameter selections.
